# Influence of Snoring on the Incidence of Metabolic Syndrome: A Community-Based Prospective Cohort Study in Rural Northeast China

**DOI:** 10.3390/jcm12020447

**Published:** 2023-01-05

**Authors:** Shasha Yu, Xiaofan Guo, Guangxiao Li, Hongmei Yang, Yingxian Sun

**Affiliations:** 1 Department of Cardiology, First Hospital of China Medical University, 155 Nanjing North Street, Heping District, Shenyang 110001, China; 2 Department of Clinical Epidemiology, Institute of Cardiovascular Diseases, First Hospital of China Medical University, 155 Nanjing North Street, Heping District, Shenyang 110001, China

**Keywords:** snoring, metabolic syndrome, rural, incidence

## Abstract

In recent years, there has been an increase in the incidence of metabolic syndrome (MetS) in rural China. Thus, for better intervention, it is necessary to identify the possible risk factors of MetS. This community-based prospective cohort study was performed to evaluate the relationship between the snoring status and incidence of MetS. In this Northeast China rural cardiovascular health study, 4980 residents aged ≥35 years (2586 men and 2394 women; follow-up proportion: 87.5%) at baseline were recruited between 2012 and 2013 and were followed up between 2015 and 2017. The primary outcome was the incidence of MetS, as defined by the unified criteria for MetS defined in 2009. The residents were classified based on their snoring status, and the outcomes were compared between the two groups. The odds ratio (OR) for MetS incidence was estimated using a logistic regression model and adjusted for confounding factors. With a median follow-up duration of 4.6 years, the MetS incidence was higher among the snorers (men, 26.2%; women, 33.5%) than in the non-snorers (men, 19.7%; women, 23.2%). The participants’ diastolic blood pressure was increased at follow-up as compared with the baseline values among the male snorers; however, a decrease was noted among the male non-snorers. Similarly, the female snorers had higher blood glucose levels during the follow-up, whereas the non-snorers had lower blood sugar levels. A significant association was noted between snoring and the incidence of MetS (adjusted OR = 1.51; 95% confidence interval = 1.32–1.74). Moreover, the incidence of severe snoring increased with increased levels of snoring, with severe snorers having an OR twice as high as that of the non-snorers (adjusted OR = 2.10; 95% confidence interval = 1.38–3.20). Overall, snoring was independently associated with a higher incidence of newly diagnosed MetS in rural Northeast China. Thus, more attention should be paid to residents with snoring problems.

## 1. Introduction

Sleep disorders are prevalent among both adults and children worldwide and are known to worsen with age [1,2,3]. Snoring is a precursor to obstructive sleep apnoea (OSA), which is the most serious sleep disorder [4]. Previous studies have shown that sleep disorders and snoring are associated with many metabolic disorders, including hypertension, diabetes, and cardiovascular diseases, such as coronary heart disease, stroke, or even cancer [5,6,7]. However, data on the association between snoring and metabolic syndrome (MetS) are conflicting. In addition, most of the previous studies were cross-sectional and performed in well-developed regions. Prospective studies are particularly limited in rural China. MetS is a combination of abdominal obesity, hyperlipidaemia, elevated blood pressure, and insulin resistance and confers a higher risk of cardiovascular events and mortality [8,9]. People that live in rural China have their unique way of life and habits.

Our previous study reported a relatively high prevalence of metabolic diseases, such as hypertension (51.1%; 53.9% in men and 48.7% in women), diabetes (10.0% in men and 11.1% in women), and dyslipidaemia (36.9%), among residents from rural Northeast China [2,10]. However, it remains unclear whether risk factors other than smoking, drinking, and unhealthy diet patterns can affect the incidence of MetS among rural residents. Therefore, it is necessary to confirm whether snoring plays a role in MetS development among rural Chinese residents. Furthermore, to address these metabolic disorders, strategies such as diet and lifestyle regulation, health education propagation, and the administration of medical treatment have been adopted. We also demonstrated that village doctor-led interventions, including the propagation of health-related knowledge, recommendation of healthy lifestyle habits, and monitoring of the participants’ blood pressure (BP), have caused significant improvements in BP control among hypertensive rural Chinese residents [11]. This highlights that effective interventions can significantly alleviate metabolic disorders. However, many risk factors, e.g., snoring status, that have not received enough attention still exist.

A few rural residents were concerned about their sleep situation and snoring status. Therefore, we hypothesised that compared with non-snorers, snorers suffer from an independently higher risk of MetS. Hence, in the present study, we aimed to validate the possible association between the snoring status and MetS incidence.

## 2. Methods

### 2.1. Study Design and Data Source

**Figure 1:**
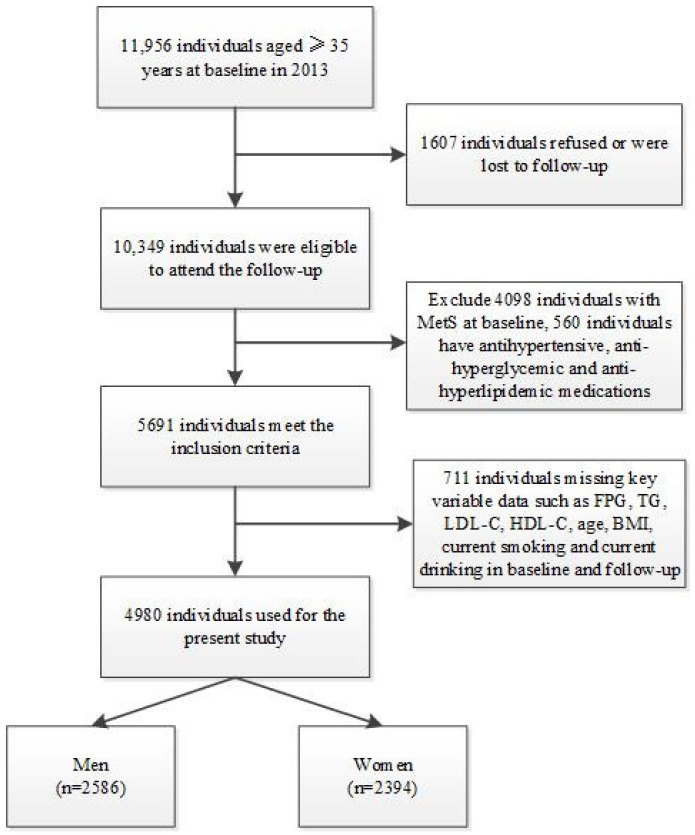
Flow chart of the participants included in this study after inclusion and exclusion.

The Northeast China rural cardiovascular health study discussed in this paper is a prospective community-based cohort study that was conducted in rural Northeast China. The design and inclusion criteria of the study have been described previously [2,12]. Between 2012 and 2013, a total of 11,956 participants aged >35 years were recruited from Dawa, Zhangwu, and Liaoyang counties, Liaoning province. Using a randomised stratified cohort sample, detailed baseline patient information was collected. The participants were invited for follow-ups in 2015 and 2017. Of the 11,956 participants, 1256 were excluded due to a lack of contact information, and 10,349 (86.6%) participants completed at least 1 follow-up visit. The study was approved by the Ethics Committee of the China Medical University (Shenyang, China). Written informed consent was obtained from all of the participants before study initiation. The detailed participant inclusion process is outlined in Figure 1.

### 2.2. Study Variables

At the outset, standardised questionnaires were used to collect details on the patients’ demographic characteristics, diet and lifestyle-related factors, and medical history [12]. The current use of tobacco and alcohol was defined. Regular exercise was defined in the questionnaire with the question “whether you exercise regularly”, with the answers yes = 0 and no = 1. The results of the snoring assessments were collected from the residents and their bed partners or family members. The snoring intensity was graded as mild (louder than breathing) (603, 12.1%), moderate (similar to talking) (572, 11.5%), severe (louder than talking) (371, 7.4%), and very severe (very noisy, could be heard in the next room) (102, 2.1%). Self-reported sleep durations (including nocturnal and nap durations) were obtained from the questionnaire. The total sleep time was defined as the total hours of sleep in 24 h. Weight and height were measured with the participants wearing light clothing and no shoes. A non-elastic tape was used to measure the waist circumference at the umbilicus. The participants’ body mass index (BMI) was calculated using the following formula: BMI = mass (kg)/height^2^ (m). Obesity was defined by a BMI of ≥28 kg/m^2^ [13]. Using a standardised automatic electronic sphygmomanometer (HEM-907; Omron, Tokyo, Japan), the participants’ BP was measured three times, with the participants seated after at least 5 min of rest. Hypertension was defined by a systolic BP (SBP) of >140 mm Hg and/or diastolic BP (DBP) of >90 mm Hg, as well as by the use of antihypertensive medications [14]. Fasting blood samples were collected from those who had fasted for at least 12 h in the morning. Enzymatic analysis was performed to assess the participants’ fasting plasma glucose, total cholesterol (TC), low-density lipoprotein cholesterol, high-density lipoprotein cholesterol (HDL-C), triglyceride (TG), serum creatinine, and other routine blood biochemical levels. The Chronic Kidney Disease Epidemiology Collaboration equation was used to calculate the estimated glomerular filtration rate (eGFR) [15]. MetS was diagnosed following the unified criteria for MetS defined in a 2009 meeting between several major organisations [8]. Based on these criteria, MetS was diagnosed when at least three of the following five risk factors were present: (1) elevated waist circumference (defined by the population and country), 90 cm for men and 80 cm for women (Asians, Japanese, and South and Central Americans); (2) elevated triglyceride levels, 150 mg/dL (1.7 mmol/L; drug treatment for elevated triglycerides is an alternate indicator); (3) low HDL-C levels, 40 mg/dL (1.0 mmol/L) among men and 50 mg/dL (1.3 mmol/L) among women (drug treatment for low HDL-C is an alternative indicator); (4) elevated BP levels, 130 mm Hg systolic and/or 85 mm Hg diastolic (another indicator is antihypertensive drug treatment in patients with a history of hypertension); and (5) elevated fasting glucose levels, 100 mg/dL (drug treatment for elevated glucose levels is an alternate indicator).

### 2.3. Statistical Analysis

Descriptive statistics were calculated for all of the variables, including continuous (indicated as mean values and standard deviations) and categorical (indicated as numbers and percentages) variables. The residents were categorised into the non-snorer (n = 3332) or snorer (n = 1640) group. Changes in the body weight, BMI, and other parameters at baseline and follow-up between the two groups were compared using Student’s *t*-test or the Wilcoxon rank sum test. Logistic regression analyses were used to estimate the odds ratio (ORs) and 95% confidence intervals (CIs) in the analysis of the association between snoring and MetS incidence after adjusting for possible confounders. The logistic regression model included the following variables: age (continuous), snoring status (non-snorer/snorers), sleep duration (continuous), eGFR (continuous), regular exercise (yes/no), current smoking status (yes/no), and current drinking status (yes/no). All the statistical data were analysed using the SPSS software version 20.0 (SPSS Inc., Chicago, IL, USA), and *p* < 0.05 was considered as statistically significant.

## 3. Results

The baseline characteristics of the 4980 residents (2586 men and 2394 women; follow-up proportion: 87.5%; follow-up duration: 4.6 years) are presented in Table 1. Among the women, their weight, BMI, waist circumference, SBP, DBP, TC, TG, HbA1c, and low-density lipoprotein cholesterol were higher and their eGFR was lower at baseline in the snorers than in the non-snorers; by contrast, among the men, only their weight, BMI, waist circumference, and DBP were higher in the snorers than in the non-snorers. Moreover, the rate of current drinkers was significantly higher among male snorers, whereas the rate of current smokers was higher among the female snorers.

Table 2 shows the changes in the patients’ clinical characteristics at baseline and follow-up stratified by snoring status. Among men, their DBP was greatly increased at the follow-up for the snorers, but decreased for the non-snorers. A relatively smaller decrease in SBP was noted in the snorers than in the non-snorers at follow-up. As for women, a significant increase in their fasting blood glucose (FBG) levels was noted at follow-up in the snorers; however, compared with that at baseline, the FBG decreased at follow-up in the non-snorers. For both sexes, a higher decrease in current smokers from the follow-up to baseline was noted among the snorers compared to the non-snorers.

The subgroup analysis revealed differences between both the male and female snorers and non-snorers. Among the males, compared with the non-snorers, the snorers only had a higher incidence of hypertension (63.4% vs. 59.4%; *p* = 0.019), abdominal obesity (33.1% vs. 22.1%; *p* < 0.001), and MetS (26.2% vs. 19.7%; *p* < 0.001). Among the females, compared with the non-snorers, the snorers had a higher incidence of MetS (33.5% vs. 23.2%; *p* < 0.001), abdominal obesity (62.1% vs. 45.6%; *p* < 0.001), hypertension (47.5% vs. 39.4%; *p* < 0.001), hyperglycaemia (27.3% vs. 21.0%; *p* < 0.001), and high TG (27.6% vs. 21.6%; *p* = 0.002) (Table 3).

**Figure 2:**
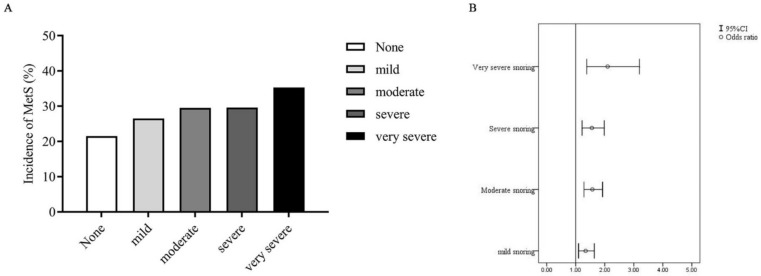
(**A**) Incidence of MetS among different snoring groups. (**B**) Odds ratio and 95% CI for MetS among different snoring groups.

The crude proportion of MetS incidence was significantly higher in the snorers (men: 26.2% (271/106); women: 33.5% (205/612)) than in the non-snorers (men: 19.7% (305/1550); women: 23.2% (413/1782)). Logistic regression analysis estimated a significant association between the snoring status and MetS incidence after adjusting for possible confounders such as age, baseline clinical characteristics, and lifestyle (adjusted OR (95% CI): 1.51 (1.32–1.74); Table 4). An additional sex-based subgroup analysis was performed, which revealed that the significant association between the snoring status and MetS incidence persisted among both men and women (adjusted OR (95% CI): 1.43 (1.19–1.73) for men and 1.505 (1.23–1.85) for women; Table 5). As shown in Figure 2A, an increase in the degree of snoring simultaneously increased the MetS incidence (26.5% for mild; 29.5% for moderate; 29.6% for severe; 35.3% for very severe). As shown in Figure 2B, snoring intensity is also correlated with MetS.

**Figure 3:**
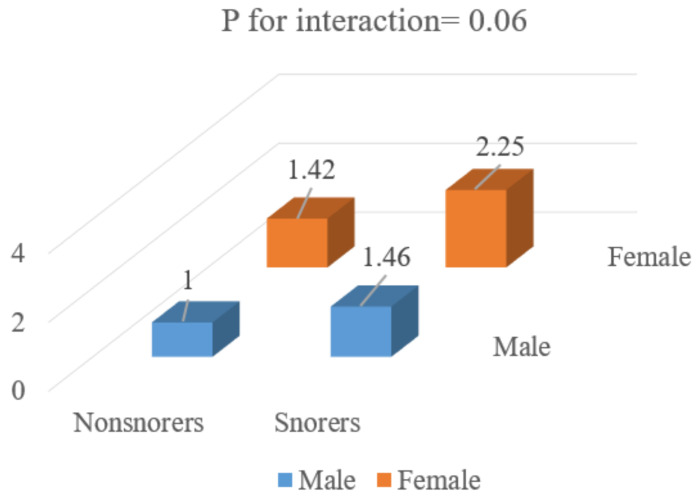
ORs for MetS according to gender and snoring status. ORs according to joint classification were adjusted for age, sleep duration, exercise, eGFR and current smoking and drinking status.

The association between sex and snoring had no effects on the MetS incidence, as observed through joint classification analysis (*p* for interaction = 0.063) (Figure 3).

Another subgroup analysis based on the participants’ snoring degree also revealed a significant association between the snoring degree and MetS incidence (adjusted OR (95% CI): 1.34 (1.10–1.64) for mild; 1.57 (1.29–1.92) for moderate; 1.56 (1.22–1.98) for severe; 2.10 (1.38–3.20) for very severe).

## 4. Discussion

Previous studies have revealed the association between snoring and MetS [7,16,17,18]. To our knowledge, most of these studies were cross-sectional studies that were unable to draw any causal inferences. Furthermore, none of these studies enrolled subjects from rural regions. The present study is a prospective cohort study that documents the effects of snoring on MetS incidence in rural China. We confirmed that snorers with an average follow-up of 4.6 years had a significantly higher MetS incidence than non-snorers. In addition, increased levels of snoring led to an increased MetS incidence. The odds ratios for very severe snorers were double those of non-snorers. The participants’ SBP decreased less dramatically at the follow-up among the male snorers in comparison to the non-snorers. Compared to the non-snorers, the male and female snorers also reported significant increases in their DBP and FBG at follow-up. Taken together, snoring may contribute to a higher risk of mental stress; thus, more attention should be paid to sleep disorders, especially in residents of rural areas.

MetS reportedly increases the risk of cardiovascular diseases. Various factors, including diet habits, sedentary lifestyles, and smoking and drinking habits, can contribute to the development of MetS. Early detection and intervention can help to alleviate these factors; by contrast, symptoms of sleep disorders, especially snoring, are less of a concern. This study reported a high rate of self-reported snoring (31.1%) at baseline in rural Northeast Chinese residents. This number was higher than that reported in most previous studies (14.14% in the Fujian Province in southeast China and 15.5% in the Guangdong Province in southern China) [7,19]. Through this prospective community-based cohort study, our data confirm the strong association between the snoring status and MetS incidence among both men and women that reside in rural areas. The association between snoring and MetS has also been proven in many previous studies [16,17,18]. A clear dose–response relationship between an increased frequency of snoring and increased incidence of each metabolic component was reported among adult Korean men and women [20]. In rural Korean communities, an increasing trend of ORs for the MetS of different snorers was reported (OR = 1.42 for rare snoring; 1.79 for occasional snoring; 2.03 for habitual snoring) [16]. An elevated trend based on the snoring frequency in the prevalence of metabolic disorders among residents from southeast China was also noted. This association remained significant even after the adjustment for possible confounding factors [7]. Another study has reported an association between snoring and hypertension [21,22,23,24]. Our data suggest that male snorers had a relatively smaller decrease in SBP. As for DBP, a significantly increasing trend was reported in the snorers. Among the female residents, the participants’ FBG decreased in the non-snorers, but increased in the snorers. A prospective study that enrolled 69,852 American women without diabetes at baseline diagnosed 1957 of these women with type II diabetes after 10 years of follow-up; further analysis revealed that snoring was associated with the risk of diabetes, after adjusting for possible confounders [25]. Hence, the higher MetS incidence among female snorers might be related to the increasing trend of FBG. Therefore, snorers should address the presence of metabolic disorders in their routine physical examinations. In particular, special attention should be paid to the possibility of hyperglyceridaemia in snorers. The mechanism that underlies the association between snoring and metabolic disorders is not yet fully understood. One possible reason might be the intermittent hypoxia and sleep deprivation caused by snoring, inducing sympathetic nervous activation, chronic inflammation, and oxidative stress, all of which increase the risk of insulin resistance and elevate one’s BP, thereby causing MetS [26,27].

This study has some limitations that need to be addressed. First, we used self-reported questionnaires rather than objective measurements such as polysomnography, leaving room for misclassification. For example, some participants who lived alone may have answered ‘no’ to the question about snoring, despite being snorers or even strong snorers. These discrepancies may cause bias in the association between snoring and MetS. However, many previous studies have already reported the association between self-reported snoring and various clinical outcomes [25,28]. Although errors in the analysis could be reduced through precise clinical measures, these measures are unavailable for large epidemiological studies. Second, snoring is the major symptom of OSA and is correlated with excessive daytime sleepiness; however, we did not evaluate these two variables in the present study. We also did not measure OSA in this study; thus, the health effects of simple snoring could not be determined. Third, the variables assessed using the questionnaire, such as sleep duration, smoking status, current drinking status, and exercise patterns, might present recall bias. Fourth, unmeasured confounding variables could have affected the association between snoring and MetS in this study. Finally, we only enrolled rural residents from Northeast China; therefore, the sample diversity is insufficient.

## 5. Conclusions

Taken together, we confirmed the increasing MetS incidence among snorers compared to non-snorers. Snoring may serve as a symptom that can help rural doctors to identify subjects at a high risk of MetS and to recommend proper screening or prevention strategies to avoid illnesses. Therefore, it is necessary to assess patients’ sleep patterns, especially their snoring status, for the early detection of MetS. Besides lifestyle adjustment, more attention should be paid to the management of snoring.

## Figures and Tables

**Table 1:** Baseline characteristics of subjects with or without snoring ^i^.

	Men		Women	
	Non-Snorers	Snorers	Non-Snorers	Snorers
**Participants, n**	1550	1036	1782	612
**Incidence of MetS**	**305 (19.7)**	**271 (26.2)**	**413 (23.2)**	**205 (33.5)**
**Age, years**	54.51 ± 11.03	53.88 ± 9.78	**50.12 ± 9.61**	**53.21 ±8.97**
**Height, (cm)**	165.75 ± 6.47	166.13 ± 6.45	155.82 ± 6.08	155.46 ± 6.44
**Weight, kg**	**64.21 ± 8.60**	**66.76 ± 9.16**	**56.38 ± 8.67**	**58.65 ± 9.84**
**BMI ^a^ (kg/m^2^)**	**23.37 ± 2.94**	**24.19 ± 3.13**	**23.20 ± 3.23**	**24.25 ± 3.67**
**Waist circumference (cm)**	**79.51 ± 7.71**	**81.27 ± 8.15**	**75.68 ± 8.04**	**78.59 ± 9.08**
**SBP ^b^ (mmHg)**	139.60 ± 21.97	140.36 ± 21.63	**131.93 ± 20.83**	**135.80 ± 22.36**
**DBP ^c^ (mmHg)**	**81. 01 ± 10.87**	**82.28 ± 11.36**	**77.42 ± 10.46**	**78.91 ± 10.88**
**FPG ^f^ (mmol/L)**	5.61 ± 1.14	5.65 ± 1.25	5.37 ± 0.90	5.39 ± 0.82
**TC ^g^ (mmol/L)**	5.07 ± 0.98	5.12 ± 0.94	**5.07 ± 1.00**	**5.28 ± 1.05**
**TG ^h^ (mmol/L)**	1.18 ± 0.99	1.17 ± 0.69	**1.08 ± 0.58**	**1.15 ± 0.50**
**HDL-C ^d^ (mmol/L)**	1.52 ± 0.42	1.50 ± 0.43	1.54 ± 0.34	1.54 ± 0.34
**LDL-C ^e^ (mmol/L)**	2.80 ± 0.74	2.85 ± 0.74	**2.79 ± 0.76**	**2.95 ± 0.83**
**eGFR (mL/min/1.73 m^2^)**	95.61 ± 12.75	96.18 ± 14.82	**95.59 ± 14.82**	**93.69 ± 14.73**
**HbA1c**	5.22 ± 0.85	5.31 ± 0.90	**5.16 ± 0.64**	**5.32 ± 0.59**
**Sleep time (hours)**	7.38 ± 1.62	7.47 ± 1.56	7.12 ± 1.69	7.10 ± 1.75
**Current smoker (%)**	58.6	60.5	**14.8**	**21.1**
**Current drinker (%)**	**43.5**	**51.4**	2.6	3.9
**Regular exercise (%)**	19.2	18.8	17.3	19.6

Data are shown as means ± SD, percentages or absolute numbers. All values in parentheses represent the standard deviation. Bold means *p* < 0.05. ^a^ Body mass index, ^b^ systolic blood pressure, ^c^ diastolic blood pressure, ^d^ high-density lipoprotein cholesterol, ^e^ low-density lipoprotein cholesterol, ^f^ fasting plasma glucose, ^g^ total cholesterol; ^h^ triglycerides. ^i^ *p* values were calculated by *t*-test (continuous variables), Wilcoxon rank sum test (continuous variables), or chi-squared test (categorical variables).

**Table 2:** Changes in clinical characteristics with or without snoring between baseline and follow up.

	Men			Women		
	Non-Snorers	Snorers	*p* Values	Non-Snorers	Snorers	*p* Values
**Participants (n)**	1550	1036		1782	612	
**Incidence of MetS (%)**	19.7	26.2	<0.001	23.2	33.5	<0.001
**△** **Weight (kg)**	0.09 ± 0.01	0.12 ± 0.01	0.893	0.33 ± 0.12	0.61 ± 0.22	0.281
**△** **BMI ^a^ (kg/m^2^)**	0.85 ± 0.07	0.92 ± 0.09	0.524	−0.38 ± 0.07	−0.34 ± 0.11	0.764
**△** **Waist circumference (cm)**	3.96 ± 0.18	4.08 ± 0.22	0.526	3.82 ± 0.18	4.35 ± 0.27	0.122
**△** **SBP ^b^ (mmHg)**	−3.68 ± 0.46	−2.04 ± 0.56	0.024	−4.58 ± 0.42	−5.07 ± 0.72	0.555
**△** **DBP ^c^ (mmHg)**	−0.45 ± 0.14	0.51 ± 0.19	0.012	−1.11 ± 0.21	−0.06 ± 0.35	0.763
**△** **FBG ^f^ (mmol/L)**	0.07 ± 0.03	0.09 ± 0.03	0.672	−0.06 ± 0.02	0.06 ± 0.04	0.003
**△** **TC ^g^ (mmol/L)**	−0.29 ± 0.02	−0.33 ± 0.03	0.336	−0.29 ± 0.02	−0.28 ± 0.03	0.895
**△** **TG ^h^ (mmol/L)**	0.24 ± 0.03	0.32 ± 0.04	0.135	0.27 ± 0.02	0.31 ± 0.03	0.306
**△** **HDL-C ^d^ (mmol/L)**	−0.08 ± 0.01	−0.10 ± 0.01	0.148	−0.08 ± 0.02	−0.08 ± 0.03	0.624
**△** **LDL-C ^e^ (mmol/L)**	0.19 ± 0.02	0.17 ± 0.02	0.501	0.22 ± 0.02	0.25 ± 0.03	0.456
**△** **eGFR (mL/min/1.73 m^2^)**	−3.24 ± 0.27	−2.62 ± 0.40	0.176	0.04 ± 0.01	0.21 ± 0.02	0.776
**△** **Current smoker (%)**	−2.4	−5.0	<0.001	−1.0	−7.3	<0.001
**△** **Current drinker (%)**	2.4	2.8	0.476	1.4	2.0	0.126

All values in parentheses represent the standard deviation. ^a^ Body mass index, ^b^ systolic blood pressure, ^c^ diastolic blood pressure, ^d^ high-density lipoprotein cholesterol, ^e^ low-density lipoprotein cholesterol, ^f^ fasting plasma glucose, ^g^ total cholesterol; ^h^ triglycerides.

**Table 3:** Incidence of MetS in non-snorers and snorers.

		Non-Snorers	Snorers	*p* Value
**Total**	**MetS**	718(21.5)	476(28.9)	<0.001
	**Abdominal obesity**	1155(34.7)	702(42.6)	<0.001
	**Hypertension**	1621(48.6)	948(57.5)	<0.001
	**High TG**	764(22.9)	450(27.3)	<0.001
	**Low HDL-C**	780(23.4)	347(21.1)	0.033
	**Hyperglycaemia**	957(28.7)	579(35.1)	<0.001
**Men**	**MetS**	305(19.7)	271(26.2)	<0.001
	**Abdominal obesity**	343(22.1)	322(31.1)	<0.001
	**Hypertension**	919(59.3)	657(63.4)	0.019
	**High TG**	379(24.5)	281(27.1)	0.070
	**Low HDL-C**	200(12.9)	134(12.9)	0.513
	**Hyperglycaemia**	583(37.6)	412(39.8)	0.144
**Women**	**MetS**	413(23.2)	205(33.5)	<0.001
	**Abdominal obesity**	812(45.6)	380(62.1)	<0.001
	**Hypertension**	702(39.4)	291(47.5)	<0.001
	**High TG**	385(21.6)	169(27.6)	0.002
	**Low HDL-C**	580(32.5)	213(34.8)	0.165
	**Hyperglycaemia**	374(21.0)	167(27.3)	0.001

MetS: metabolic syndrome; TG: triglycerides; HDL: high-density lipoprotein cholesterol.

**Table 4:** Association between snoring status and incidence of MetS.

	ORs (95% CIs)	
	Crude	Multivariate
**Snoring (ref: no)**	1.48 (1.29–1.69)	1.51 (1.32–1.74)
**Men (ref: women)**	0.82 (0.72–0.94)	0.68 (0.58–0.80)
**Age (1-year increase)**	1.02 (1.01–1.02)	1.02 (1.01–1.03)
**eGFR (1 mL/min/1.73 m^2^ increase)**	0.99 (0.98–0.99)	0.99 (0.99–1.00)
**Sleep duration (1 h increase)**	1.01 (0.97–1.05)	1.03 (0.99–1.07)
**Exercise (ref: no)**	1.17 (0.99–1.38)	1.06 (0.89–1.26)
**Current smoker (ref: never smoked or a former smoker)**	0.91 (0.80–1.04)	0.94 (0.81–1.10)
**Current drinker (ref: never drank or a former drinker)**	1.01 (0.87–1.17)	1.20 (1.00–1.44)

CI, confidence interval; OR, odds ratio.

**Table 5:** ORs (95% CIs) for incidence of MetS at follow-up (gender subgroup analysis); relationship between snoring status and incidence of MetS.

	OR (95% CI)			
	Men		Women	
	Crude	Multivariate	Crude	Multivariate
**Snoring (ref: no)**	1.45	1.43	1.67	1.51
	(1.20–1.74)	(1.19–1.73)	(1.37–2.04)	(1.23–1.85)
**Age (1-year increase)**	1.00	0.99	1.04	1.04
	(0.99–1.01)	(0.99–1.01)	(1.03–1.05)	(1.03–1.05)
**eGFR (1 mL/min/1.73 m^2^ increase)**	0.99	0.99	0.99	1.00
	(0.98–0.99)	(0.98–1.00)	(0.98–0.99)	(0.99–1.01)
**Sleep duration (1 h increase)**	1.01	1.02	1.01	1.06
	(0.96–1.07)	(0.96–1.08)	(0.96–1.07)	(1.00–1.12)
**Exercise (ref: no)**	1.04	1.01	1.33	1.16
	(0.82–1.31)	(0.79–1.29)	(1.06–1.68)	(0.91–1.47)
**Current smoker (ref: never smoked or a former smoker)**	0.85	0.81	1.29	1.07
	(0.70–1.02)	(0.67–0.99)	(1.02–1.64)	(0.83–1.37)
**Current drinker (ref: never drank or a former drinker)**	1.20	1.26	1.05	0.86
	(0.99–1.44)	(1.03–1.53)	(0.62–1.79)	(0.49–1.51)

CI, confidence interval; OR, odds ratio.

## Data Availability

Data can be provided by the corresponding author upon reasonable request.

## References

[1] Zhang X., Li X.D., Feng G.S., Xu Z.F., Du J.N., Wang G.X., Ma J., Hu P.J., Yan X.Y., Zhang J. (2019). The prevalence of snoring and its related family factors of children from 3 to 14 years old in Beijing. Zhonghua Er Bi Yan Hou Tou Jing Wai Ke Za Zhi Chin. J. Otorhinolaryngol. Head Neck Surg..

[2] Wali S.O., Abaalkhail B.A. (2015). Prevalence and predictors of habitual snoring in a sample of Saudi middle-aged adults. Saudi Med. J..

[3] Ward S.A., Pase M.P. (2020). Advances in pathophysiology and neuroimaging: Implications for sleep and dementia. Respirology.

[4] Collop N.A. (2005). Obstructive sleep apnea syndromes. Semin. Respir. Crit. Care Med..

[5] Yunus F.M., Khan S., Mitra D.K., Mistry S.K., Afsana K., Rahman M. (2018). Relationship of sleep pattern and snoring with chronic disease: Findings from a nationwide population-based survey. Sleep Health.

[6] Li Y., Gao Q., Li L., Shen Y., Lu Q., Huang J., Sun C., Wang H., Qiao N., Wang C. (2019). Additive interaction of snoring and body mass index on the prevalence of metabolic syndrome among Chinese coal mine employees: A cross-sectional study. BMC Endocr. Disord..

[7] Huang J., Qi J., Lin Q., Li S., Chen G., Ding H., Zhao J. (2018). Snoring and components of metabolic syndrome in Southeastern Chinese adults: A community-based study. Clin. Respir. J..

[8] Alberti K.G., Eckel R.H., Grundy S.M., Zimmet P.Z., Cleeman J.I., Donato K.A., Fruchart J.C., James W.P., Loria C.M., Smith S.C. (2009). Harmonizing the metabolic syndrome: A joint interim statement of the International Diabetes Federation Task Force on Epidemiology and Prevention; National Heart, Lung, and Blood Institute; American Heart Association; World Heart Federation; International Atherosclerosis Society; and International Association for the Study of Obesity. Circulation.

[9] Eckel R.H., Grundy S.M., Zimmet P.Z. (2005). The metabolic syndrome. Lancet.

[10] Zhou X., Guan H., Zheng L., Li Z., Guo X., Yang H., Yu S., Sun G., Li W., Hu W. (2015). Prevalence and awareness of diabetes mellitus among a rural population in China: Results from Liaoning Province. Diabet. Med. J. Br. Diabet. Assoc..

[11] Sun Y., Mu J., Wang D.W., Ouyang N., Xing L., Guo X., Zhao C., Ren G., Ye N., Zhou Y. (2022). A village doctor-led multifaceted intervention for blood pressure control in rural China: An open, cluster randomised trial. Lancet.

[12] Li Z., Guo X., Zheng L., Sun Z., Yang H., Sun G., Yu S., Li W., Zou L., Wang J. (2014). Prehypertension in rural northeastern China: Results from the northeast China rural cardiovascular health study. J. Clin. Hypertens..

[13] Yu S., Yang H., Guo X., Zheng L., Sun Y. (2017). Metabolic syndrome and depressive symptoms among rural Northeast general population in China. BMC Public Health.

[14] Chobanian A.V., Bakris G.L., Black H.R., Cushman W.C., Green L.A., Izzo J.L., Jones D.W., Materson B.J., Oparil S., Wright J.T. (2003). The Seventh Report of the Joint National Committee on Prevention, Detection, Evaluation, and Treatment of High Blood Pressure: The JNC 7 report. Jama.

[15] Levey A.S., Stevens L.A., Schmid C.H., Zhang Y.L., Castro A.F., Feldman H.I., Kusek J.W., Eggers P., Van Lente F., Greene T. (2009). A new equation to estimate glomerular filtration rate. Ann. Intern. Med..

[16] Shin M.H., Kweon S.S., Choi B.Y., Kim M.K., Chun B.Y., Shin D.H., Lee Y.H. (2014). Self-reported snoring and metabolic syndrome: The Korean Multi-Rural Communities Cohort Study. Sleep Breath. Schlaf Atm..

[17] Kim C.E., Shin S., Lee H.W., Lim J., Lee J.K., Kang D. (2017). Frequency of Loud Snoring and Metabolic Syndrome among Korean Adults: Results from the Health Examinees (HEXA) Study. Int. J. Environ. Res. Public Health.

[18] Zou J., Song F., Xu H., Fu Y., Xia Y., Qian Y., Zou J., Liu S., Fang F., Meng L. (2019). The Relationship between Simple Snoring and Metabolic Syndrome: A Cross-Sectional Study. J. Diabetes Res..

[19] Thomas G.N., Jiang C.Q., Lao X.Q., McGhee S.M., Zhang W.S., Schooling C.M., Adab P., Lam T.H., Cheng K.K. (2006). Snoring and vascular risk factors and disease in a low-risk Chinese population: The Guangzhou Biobank Cohort Study. Sleep.

[20] Cho N., Joo S., Kim J., Abbott R.D., Kim J., Kimm K., Shin C. (2006). Relation of habitual snoring with components of metabolic syndrome in Korean adults. Diabetes Res. Clin. Pract..

[21] Goto R., Tanigawa T., Maruyama K., Tomooka K., Eguchi E., Osawa H., Saito I. (2020). Associations of snoring frequency with blood pressure among the lean Japanese population: The Toon Health Study. J. Hum. Hypertens..

[22] Hoffstein V. (1994). Blood pressure, snoring, obesity, and nocturnal hypoxaemia. Lancet.

[23] Hoffstein V., Mateika J. (1992). Evening-to-morning blood pressure variations in snoring patients with and without obstructive sleep apnea. Chest.

[24] Furukawa T., Nakano H., Yoshihara K., Sudo N. (2016). The Relationship between Snoring Sound Intensity and Morning Blood Pressure in Workers. J. Clin. Sleep Med. JCSM.

[25] Al-Delaimy W.K., Manson J.E., Willett W.C., Stampfer M.J., Hu F.B. (2002). Snoring as a risk factor for type II diabetes mellitus: A prospective study. Am. J. Epidemiol..

[26] Sun L., Pan A., Yu Z., Li H., Shi A., Yu D., Zhang G., Zong G., Liu Y., Lin X. (2011). Snoring, inflammatory markers, adipokines and metabolic syndrome in apparently healthy Chinese. PLoS ONE.

[27] Fava C., Montagnana M., Favaloro E.J., Guidi G.C., Lippi G. (2011). Obstructive sleep apnea syndrome and cardiovascular diseases. Semin. Thromb. Hemost..

[28] Wang T., Lu J., Wang W., Mu Y., Zhao J., Liu C., Chen L., Shi L., Li Q., Yang T. (2015). Sleep duration and snoring associate with hypertension and glycaemic control in patients with diabetes. Diabet. Med. J. Br. Diabet. Assoc..

